# The optimization of electrochemical immunosensors to detect epithelial sodium channel as a biomarker of hypertension

**DOI:** 10.5599/admet.1629

**Published:** 2023-02-17

**Authors:** Tias F.H. Lestari, Riyanto Setiyono, Nina Tristina, Yulia Sofiatin, Yeni Wahyuni Hartati

**Affiliations:** 1 Department of Chemistry, Faculty of Mathematics and Natural Sciences, Universitas Padjadjaran, Indonesia; 2 Department of Clinical Pathology, Faculty of Medicine, Universitas Padjadjaran, Indonesia; 3 Department of Public Health, Faculty of Medicine, Universitas Padjadjaran, Indonesia

**Keywords:** Box-Behnken design, screen-printed carbon electrode, immunosensor, hypertension

## Abstract

The epithelial sodium channel (ENaC) is a transmembrane protein that regulates the balance of sodium salt levels in the body through its expression in various tissues. The increase in sodium salt in the body is related to the expression of ENaC, thereby increasing blood pressure. Therefore, overexpression of the ENaC protein can be used as a biomarker for hypertension. The detection of ENaC protein using anti-ENaC in the biosensor system has been optimized with the Box-Behnken experimental design. The steps carried out in this research are screen-printed carbon electrode modification with gold nanoparticles, then anti-ENaC was immobilized using cysteamine and glutaraldehyde. Optimum conditions of the experiment, such as anti-ENaC concentration, glutaraldehyde incubation time, and anti-ENaC incubation time, were optimized using the Box-Behnken experimental design to determine the factors that influence the increase in immunosensor current response and the optimum conditions obtained were then applied to variations in ENaC protein concentrations. The optimum experimental conditions for anti-ENaC concentration were 2.5 μg/mL, the glutaraldehyde incubation time was 30 minutes, and the anti-ENaC incubation time was 90 minutes. The developed electrochemical immunosensor has a detection limit of 0.0372 ng/mL and a quantification limit of 0.124 ng/mL for the ENaC protein concentration range of 0.09375 to 1.0 ng/mL. Thus, the immunosensor generated from this study can be used to measure the concentration of normal urine samples and those of patients with hypertension.

## Introduction

Hypertension is the number one cause of death in the world and in Southeast Asia, where Indonesia is one of the countries in the region [1]. The World Health Organization [2] explains that as many as 1.13 billion people worldwide suffer from hypertension. The number of people suffering from hypertension continues to increase every year. Based on diagnostic data in the Basic Health Research [3] conducted by doctors, it was found that the prevalence of hypertension in Indonesia in adolescents aged 18 years was 34.1%, while in the 31-44 year age group, it was 31.6 %, in the age group 45-54 years 45.3 %, and 55-64 years 55.2 %. In addition, there are other tools used for measuring blood pressure, namely the sphygmomanometer, but in its use, it requires supporting tests [4]. Hypertension can be detected through biomarkers (biological markers) in the human body. Salt-sensitive hypertension is a type of hypertension that can occur due to increased blood pressure driven by excess salt intake or salt load and due to abnormal sodium excretion function in the kidneys [5,6]. The epithelial sodium channel (ENaC) is widely expressed in tissues such as the kidney, colon, lung, intestine, sweat and salivary glands [7]. The kidneys play a role in balancing the sodium content in the body. In the kidney, precisely in the distal nephron, the total sodium (Na^+^) balance in the body is regulated through ENaC. Unbalanced sodium levels in the distal nephron can increase extracellular fluid volume, which causes an increase in blood pressure [8,9]. ENaC is located in apically polarized cell membranes that function as mediators of Na^+^ transport across the renal epithelium [9]. Urine is a substance excreted by the kidneys, so ENaC protein can be detected using a urine sample.

Sofiatin and Rusli [10] conducted a study to detect ENaC protein in human urine samples using the enzyme-linked immunosorbent assay (ELISA) method. The results of the study showed ENaC protein levels in the urine were normal, hypertension without a history, and with a history of 1.12, 4.0 and 2.7 ng/mL. However, the ELISA method has several drawbacks, including requiring trained medical personnel, a long processing time, and complicated procedures, so other methods are needed for ENaC protein in human urine. There is another method that can be used to detect ENaC protein, namely electrochemical immunosensor [11], including the research of Hartati *et al.* [12] electrochemical immunosensor study based on gold nanoparticles-anti-ENaC bioconjugate, voltammetric immunosensor using a screen-printed carbon electrode modified with reduced graphene oxide [13], and electrochemical immunosensor study based on a modified screen-printed carbon electrode with gold nanoparticles-MPA-EDC-NHS [14]. In addition, the ENaC protein detection method can also be carried out with an electrochemical aptasensor [14], who used ceria electrodeposition and Fazrin *et al.* [15] study, who used a screen-printed carbon electrode modified with CeO2/AuNP-streptavidin-biotinylated aptamer. There is also research using an optical-based biosensor for the detection of ENaC protein by Garcia Rubio *et al.* [16].

Biosensor is an analytical tool that has been widely developed, which is a tool that combines the use of biological elements such as enzymes, cells, and antibodies with a transducer to convert biological interactions into a readable output signal [17–21]. Biosensors are useful in the fields of medicine, environmental diagnostics and the food and beverage industry [20]. Electrochemical immunosensor is a type of biosensor that can detect an analyte based on the interaction between antibody-antigen through electrochemical transduction [22]. Screen-printed carbon electrode (SPCE) is an electrochemical device that is widely used in sensor applications [23-25] because it has several advantages, including low background current and a wide potential window, and does not require expensive costs [26]. The SPCE consists of three electrodes, namely a working electrode, a counter electrode and a reference electrode located on the same platform and can be made simultaneously with the screen printing technique [27]. Increasing the signal sensitivity of an immunosensor device can be done by modification of nanoparticles, such as gold nanoparticles [22]. Gold nanoparticle-modified SPCE is widely used because of its great biocompatibility [28] so biomolecules can be immobilized stably. In addition, gold nanoparticles can also increase the electroactive area of the electrode, thereby providing many active areas for protein binding and electron transfer, and are better at maintaining protein bioactivity [29]. One of the biomolecular immobilization techniques that can be done is a self-assembled monolayer [30]. A self-assembled monolayer is an ordered molecular assembly formed spontaneously by chemical adsorption and self-organization of long chains of molecules on a suitable substrate surface [31].

To identify the factors that influence the experimental results, to minimize uncontrollable factors and evaluate their influence, experiments were carried out using an experimental design [32]. Response surface methodology (RSM) is a combination of statistical and mathematical techniques used in modeling and problem analysis to determine the variables in order to optimize the response [33]. One type of experimental design from the response surface methodology is the Box-Behnken experimental design, a three-level design that matches the response surface [34]. The principle of this experimental design is based on the number of experiments carried out, the number of parameters, and the number of central points, as well as at three levels/levels for each factor, namely -1 (low), 0 (medium), +1 (high). The Box-Behnken design is more efficient because it only performs 15 experiments. The Box-Behnken experimental design is a three-level design method used to estimate the interactions that occur, carried out by maximizing and minimizing the response. The resulting designs are generally very efficient in terms of the number of rounds required [35]. To determine the optimal conditions in the Box-Behnken experimental design, a second-order polynomial model was used to fit the parameter and response relationship. The polynomial equations obtained are [36]:


(1)

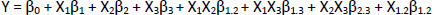



where Y = response, X_i_ = parameter that affects response (i = 1, 2, …, n) and βi = regression coefficient (i = 1, 2, …, n).

In this study, an electrochemical immunosensor method was developed for the detection of ENaC protein using Au-modified SPCE, cysteamine, glutaraldehyde, and anti-ENaC. On the surface of SPCE/Au, cysteamine is added to form a self-assembled monolayer via a thiol group (-SH). Glutaraldehyde has two terminal aldehyde groups (-CHO) which covalently bind to the free amine group (-NH_2_) of cysteamine. To increase the selectivity of the immunosensor, anti-ENaC was added to SPCE modified by gold nanoparticles, cysteamine, and glutaraldehyde. Then the Box-Behnken experimental design was used to optimize several factors that affect this electrochemical immunosensor experiment, including the concentration of anti-ENaC, glutaraldehyde incubation time, and anti-ENaC incubation time. Optimum conditions of these factors are required to obtain as much attachment of the bioreceptors to the modified electrode as possible. The detection and quantification limits were determined.

## Experimental

### Tools

The equipment used in this study were autoclave sterilizer (Hirayama Autoclave HVE-50), hot plate (IKA C-MAG HS 7), magnetic stirrer (Eppendorf), micro pipette (Eppendorf), potentiostat Zimmer and Peacock connected to a computer using PSTrace 5.8 software, centrifugator (Eppendorf), UV-Vis spectrophotometer (Thermo Scientific), scanning electron microscope (Hitachi TM 3000, Japan), homemade Screen-Printed Carbon Electrode (SPCE) produced at the Department of Chemistry, Padjadjaran University, micro tube (Eppendorf), and micro pipette tips, as well as all glass equipment in the Laboratory of the Department of Chemistry, Padjadjaran University.

### Materials

The materials used in this study were demineralized water (PT Ikapharmindo Putramas Indonesia), anti-ENaC antibody (Santa Cruz Biotechnology), hydrochloric acid (Merck; p.a), chloroauric acid (Sigma Aldrich), ethanolamine (Merck.pa), glutaraldehyde (Sigma Aldrich), potassium ferricyanide (Sigma Aldrich), potassium chloride (Merck.pa), sodium hydroxide (Merck.pa), phosphate buffered saline pH 7.4 (Merck *p.a.*), ENaC protein (Santa Cruz Biotechnology), cysteamine (Sigma Aldrich).

Preparation of 1 % trisodium citrate dihydrate solution: 0,1 g of trisodium citrate dihydrate (Na_2_C_6_H_5_O_7_.2H_2_O) was dissolved with 10 mL of demineralized water, then homogenized.

Preparation of 0.75 mM 30 mL gold nanoparticle (AuNP) solution from chloroauric acid: 268.53 μL of HAuCl_4_×3H_2_O solution was added with 19.731.4 μL of demineralized water, then stirred using a magnetic stirrer and heated using a hot plate until boiling. Then 1730 μL of 1 % Na_2_C_6_H_5_O_7_.2H_2_O was added and stirred until it changed color (yellow-colorless-purple-burgundy).

Preparation of phosphate buffer saline (PBS) solution pH 7.4: 1 PBS tablet was dissolved in 100 mL of demineralized water and stirred until homogeneous. Then the pH of the solution was adjusted by adding 0.1 M NaOH or HCl solution and measured using a pH meter to show a pH of 7.4.

Preparation of 0.01 M K_3_[Fe(CN)_6_] solution in 0.1 M KCl: K_3_[Fe(CN)_6_] was weighed as much as 0.8233 grams, then KCl was weighed as much as 1.8639 grams. The solid potassium ferricyanide and potassium chloride were dissolved in 250 mL of demineralized water.

Preparation of 0.1 M cysteamine solution: The cysteamine solid was weighed as much as 0.7715 g. Then put into a 100 mL volumetric flask and dissolved with demineralized water up to the mark and shaken until homogeneous.

Preparation of 2.5 % glutaraldehyde solution: 5 mL of 50 % glutaraldehyde dissolved in phosphate buffer saline pH 7.4 to 100 mL.

Preparation of 50 mL 1 M ethanolamine solution: 3.08 mL of 98 % ethanolamine solution was diluted with demineralized water in a 50 mL volumetric flask, and then the solution was shaken until homogeneous.

### Screen-printed carbon electrode (SPCE) modification with gold particles (SPCE-Au) and SPCE-Au characterization

SPCE electrode modification is done through the spray coating technique. Prior to the spray coating technique, the SPCE electrode was activated by irradiating it with ultraviolet light for 15 minutes. After the SPCE electrode was activated, a spray coating technique was applied using a solution of gold nanoparticles (AuNP) 10 times sprayed and dried. Then rinsed using 40 μL of demineralized water and dried at room temperature. Then, SPCE that had been modified by gold nanoparticles was characterized by differential pulse voltammetry using a 0.01 M K_3_[Fe(CN)_6_] redox system in 40 μL 0.1 M KCl over a potential range of -1.0 to +1.0 at a scan rate of 0.008 V/s for 3-4 minutes. SPCE surfaces before and after modified gold nanoparticles were observed by SEM (Scanning Electron Microscope) and characterized using electrochemical impedance spectroscopy (EIS).

### Anti-ENaC immobilization on electrode surface and characterization

The SPCE-Au surface was rinsed with demineralized water and dried at room temperature before adding 40 μL of 0.1 M cysteamine solution and incubating at room temperature for 2 hours in the dark, followed by rinsing with demineralized water [13]. Next, the sensor was covered with 2.5 % glutaraldehyde solution for 30 minutes to form a cross-linking monolayer on the SAM-modified Au electrode and washed thoroughly with demineralized water to remove unreacted glutaraldehyde [37]. Next, the electrodes were dripped with 25 L of 2.5 μg/mL anti-ENaC and incubated for 90 minutes at 25 °C to obtain SPCE-Au/Cysteamine/Glutaraldehyde/Anti-ENaC. The resulting immunosensor was characterized by differential pulse voltammetry using a 0.01 M K_3_[Fe(CN)_6_] redox system in 40 μL 0.1 M KCl in the potential range of -1.0 to +1.0 at the scanning rate. 0.008 V/s for 3-4 minutes to determine whether cysteamine, glutaraldehyde, and anti-ENaC were immobilized at the electrode. Then it was characterized using SEM and EIS [13].

### Determination of immunosensory response to ENaC

Electrodes immobilized with Anti-ENaC were incubated in 30 μL ENaC with a certain concentration for 30 minutes at 25 °C. Furthermore, at each concentration of ENaC, the current response was measured using differential pulse voltammetry to obtain SPCE-Au/Cysteamine/Glutaraldehyde/Anti-ENaC/ENaC electrodes with a redox system of 0.01 M K_3_[Fe(CN)_6_] solution in KCl 0 .1 M over a potential range of -1.0 to +1.0 V at a scan rate of 0.008 V/s for 3-4 minutes [14].

### Optimization of parameters affecting experiments

Parameter optimization was carried out using the Box-Behnken method with the concentration of anti-ENaC (X1), incubation time of glutaraldehyde (X2), and incubation time of anti-ENaC (X3), optimized through three different levels as shown in Table 1. Lowest (-1), medium (0), and highest (+1) levels. Each measurement was processed and the determination of the optimal value for each factor was carried out using the Box-Behnken experimental design on the minitab19 program.

### Calibration curve

Variations in the concentration of the ENaC solution (0, 0.09375, 0.1875, 0.375, 0.75, 1 and 1.5 ng/mL) were measured using an electrochemical immunosensor (differential pulse voltammetry with a redox system of 0.01 M K_3_[Fe(CN)_6_] in 0.1 M KCl in the potential range from -1.0 to +1.0 V at a scan rate of 0.008 V/s for 3-4 min) using the optimal conditions of the Box-Behnken method. Next, a curve is made between the concentration and the difference in the average peak current (Δ*I*) for each resulting measurement as *x* and *y*, so that the equation *y* = a + b*x* is obtained [32].

## Results and discussion

### Preparation and characterization of gold nanoparticles (AuNP)

**Figure 1.**
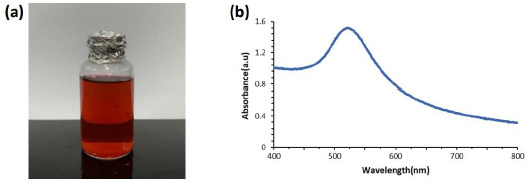
**(a)** Burgundy gold nanoparticle colloid. **(b)** the results of AuNP characterization using visible light spectrophotometer. The maximum absorption wavelength is at 521 nm.

Gold nanoparticles (AuNP) are currently widely used in immunosensor fabrication because of their high conductivity, good compatibility with biomolecules, and can increase the electroactive area of the electrode [29]. In addition, gold nanoparticles function as an electron-conducting pathway between the prosthetic group and the electrode surface and act as a catalyst for electrochemical reactions [38]. Colloidal gold nanoparticles (AuNP) were prepared with 0.75 mM chloroauric acid (HAuCl_4_.3H_2_O) solution, which was heated to boiling, then mixed with 1 % sodium citrate (Na_3_C_6_H_5_O_7_.2H_2_O) solution and vigorous stirring until the solution color changed from colorless to burgundy, as in Figure 1a.

Chlorouric acid solution acts as the main precursor in the synthesis of gold nanoparticles. Chlorouric acid solution has gold in Au^3+^ oxidation state, while gold nanoparticles have Au^0^ oxidation state [39]. Trisodium citrate solution acts as a capping agent to maintain the stability of colloidal gold nanoparticles so that they have excellent stability. Citrate can prevent aggregation of the formed gold nanoparticles [40,41]. The citrate ion has a hydroxyl (-OH) and a carboxylate (COO-) functional groups. The hydroxyl functional group on the citrate ion will interact with the reduced gold so that the gold particles will be surrounded by citrate ions (negatively charged) and prevent aggregation of the nanoparticles through the repulsion between the negative charges on the surface. The colloidal nanoparticles formed were characterized using a visible light spectrophotometer. The characterization results in Figure 1b show that the maximum absorption of gold nanoparticles is at a wavelength of 521 nm. Gold nanoparticles generally have a strong plasmon resonance absorption band at a wavelength of 510-550 nm with a spherical shape [42,43]. When gold particles are nano-sized, they can appear red, purple or blue. The color difference of the resulting gold nanoparticles comes from the surface plasmon resonance (SPR) phenomenon. When light shines on a metal surface, a surface plasmon occurs, a group of electrons that move back and forth simultaneously across the metal surface. When electrons travel at the same frequency as light, plasmons are in resonance. When plasmons are in resonance, electrons absorb and scatter light, producing the colors seen (complementary colors). Gold nanoparticles resonate at frequencies in the visible light spectrum. The smaller gold nanoparticles absorb and resonate with the blue-green light wavelengths of the spectrum (~450 nm), resulting in a red color, while the larger gold nanoparticles absorb and resonate with green-red light wavelengths, resulting in blue color. With increasing particle size, the SPR absorption wavelength shifts to the infrared region of the spectrum and the color changes according to the wavelength [41]. Based on the SPR theory, small nanoparticles have a large band gap so that the energy required to excite electrons out of the band gap is very large. The energy is inversely proportional to the wavelength, so that a small absorption of visible light wavelengths indicates a small AuNP size. Gold nanoparticles have also been characterized using a particle size analyzer (PSA) and dynamic light scattering (DLS) techniques reported in [44] with an average gold nanoparticle diameter of 38.3 nm.

### SPCE-Au modification

**Figure 2.**
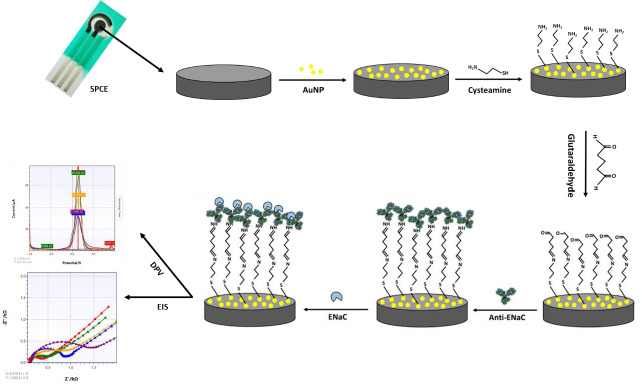
Illustration of electrochemical immunosensor for detection of ENaC protein.

Screen-printed carbon electrode (SPCE) is an electrode widely used because of its simplicity in the process, low background current, requires a small number of samples and is easily modified with gold nanoparticles. SPCE modification with gold nanoparticles aims to increase the electroactive area of the electrode. The high conductivity of gold can increase the electroactive properties of the electrode, thus providing many active regions that play a role in protein binding and electron transfer. Figure 2 shows the stages of SPCE modification using gold nanoparticles and biological elements with electrochemical characterization. In this study, the surface of the SPCE electrode was modified with gold nanoparticles (SPCE-Au) using a spray coating technique involving the formation of a fine aerosol through a nozzle [45]. The aerosol droplets will then hit the electrode surface and stick evenly to the electrode surface after being modified. Prior to spray coating, SPCE was irradiated with ultraviolet light for 15 minutes to oxidize the carbon particles so that the carbon functional groups on the SPCE surface were active. Activation of carbon shows that carbon from the relaxation energy level is excited to a higher energy level, then carbon becomes activated carbon. So that when gold nanoparticles are added, they will settle on the carbon surface. This pretreatment is also carried out to clean the electrode surface, change the microstructure and surface chemistry of the electrode, and generate new active sites that affect the sensitivity of the electrode to the target analyte [46]. SPCE before and after modification with gold nanoparticles was characterized using differential pulse voltammetry to determine whether SPCE modification with AuNP was successful. This electrochemical measurement was carried out by observing the redox activity of the electroactive indicator, namely K_3_[Fe(CN)_6_] 10 mM in 0.1 M KCl on the electrode surface. The K_3_[Fe(CN)_6_] system is used because it has a sensitive electrochemical response to carbon-based surfaces [47]. The redox reaction that occurs is the reduction of ferricyanide ions to ferrocyanide, as shown in the following reaction [48]:


(2)

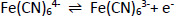



**Figure 3.**
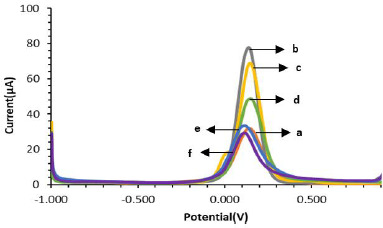
Immunosensor response using differential pulse voltammetry: (a) SPCE Bare; (b) SPCE-Au; (c) SPCE-Au/Cysteamine; (d) SPCE-Au/Cysteamine/Glutraladehyde; (e) SPCE-Au/Cysteamine/Glutaraldehyde/Anti-ENaC; and (f) SPCE-Au/Cysteamine/Glutaraldehyde/Anti-ENaC/ENaC with a redox system of 10 mM K_3_[Fe(CN)_6_] solution in 0.1 M KCl over a potential range of -1.0 to +1.0 V and scan rate 0.008 V/s.

In Figure 3 it is shown that the modified SPCE electrode is more electroactive than the unmodified electrode. This is indicated by the increase in peak current to 77.298 μA after the electrode was modified with AuNP (SPCE-Au). The peak current before modification (SPCE Bare) was 31.355 μA. The increasing peak current after modification indicates that the addition of AuNP to SPCE by spray coating technique and irradiation with UV light can increase the electron transfer process between the electrode and the analyte so that the electrode becomes more sensitive [12,49].

### Anti-ENaC immobilization on electrode surface

**Figure 4.**
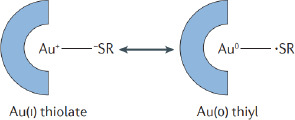
Mechanism of van der Waals dispersion forces between S-Au [51].

SPCE-Au was modified first using a self-assembled monolayer (SAM). SAM has the potential to create immobile protein layers in which some control over orientation is exerted. The formation of SAM is carried out by forming a covalent coupling of cysteamine and glutaraldehyde as a functional amino cross-linking structure. SAM can basically be used in two ways, namely, chemical modification of a protein with a linking structure that can react with a substrate to form SAM and carrying out reactions on SAM to link biomolecules [50]. SPCE-Au was incubated in cysteamine solution for 120 minutes. Cysteamine is an organic molecule that belongs to alkanethiol, can oscillate spontaneously and is stable on a gold surface. In its structure, alkanethiol consists of three parts, namely the main functional group (amine), which binds with other functional groups to produce a chemical interaction, the sulfur (S) group, which interacts with metal (Au), and the methylene group [12]. When a thiol-based solution is added to the SPCE-Au electrode, the thiol group will be chemically adsorbed onto the gold surface through the formation of a thiolate bond. The attractive van der Waals forces between the alkyl chains increase the stability and regularity of the SAM so that a strong and stable S-Au reaction will be formed. Figure 4 shows van der Waals dispersion forces mechanism between S-Au [51]:

The S-Au reaction is a Lewis acid reaction, which is a reaction based on the binding of free electrons owned by the sulfur atom (S) of cysteamine with Au. The formation of S-Au occurs through the coordination of covalent bonds. In the S-Au reaction, as many as six free electrons owned by sulfur bind to Au at the electrode surface. This reaction can be written as follows [52]:


(3)

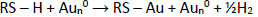



Furthermore, SPCE-Au/cysteamine, glutaraldehyde solution was added and incubated for 30 minutes. Glutaraldehyde consists of two terminal aldehyde groups (-COH), which is a bifunctional reagent because it can react with two primary amine groups, namely from biomolecules and solid surfaces [53]. Bifunctional reagents in binding biomolecules to solid media, is widely used because it facilitates the formation of a stable single layer. Anti-ENaC was immobilized on SPCE-Au/cysteamine/glutaraldehyde and incubated for 90 min. Glutaraldehyde binds to the amino acid residue lysine (positively charged), then reacts covalently with the carbonyl group of glutaraldehyde to create a more stable structure. Anti-ENaC plays an important role as a capture molecule to recognize and detect the presence of ENaC antigen in a sample.

After anti-ENaC is immobilized on the electrode surface, ethanolamine is added as a blocking agent whose presence will replace the -COH groups on the electrode surface that are not bound to the terminal amine group of anti-ENaC. This is done to prevent non-specific binding to the surface of the SPCE electrode, which can interfere with the immunoassay process. Good antibody orientation can increase the antigen-binding capacity to reduce the detection limit and increase the sensitivity to antigen. SPCE blocked with ethanolamine was then incubated with ENaC protein for 30 minutes at room temperature. After the ENaC protein was immobilized on the electrode surface, the electrode was rinsed using a PBS solution of pH 7.4 to remove unreacted or attached species to the electrode surface so that it did not interfere with the analysis process and the immunoreaction process between antibodies and antigens and minimized the measurement error of the immunosensor response. Then, characterization was carried out using DPV. The presence of the ENaC protein bound to anti-ENaC causes the ferricyanide current peak to be lower. This is because the ENaC protein is a large biomolecule that can cause the electron transfer process to be more hindered. The measured current response will be proportional to the amount of ENaC protein involved. The higher the ENaC protein concentration, the lower the current generated, while the lower the ENaC protein concentration, the higher the current generated. The higher the concentration of ENaC protein, the more ENaC protein is attached to the electrode surface.

### Characterization of SPCE-Au/cysteamine/glutaraldehyde/anti-ENaC/ENaC with differential pulse voltammetry and electrochemical impedance spectroscopy

Figure 4 shows the voltammogram of the modified SPCE characterization using differential pulse voltammetry. Characterization was carried out to determine whether anti-ENaC had been successfully modified on the SPCE surface. After the addition of cysteamine on the electrode surface to form SPCE-Au/cysteamine, the current decreased to 67.363 μA due to the formation of a strong and stable S-Au bond on the electrode surface so that the electron transfer process between ferricyanide species and the electrode was hindered. Furthermore, there was a decrease in current to 47.375 μA after the addition of glutaraldehyde, which indicated that glutaraldehyde had reacted with cysteamine on the electrode surface and blocked the electron transfer process. After the addition of Anti-ENaC again, the current decreased to 31.886 μA, this is because the ENaC protein is a large biomolecule that can hinder the electron transfer process.

SPCE-Au characterization was also carried out by electrochemical impedance spectroscopy (EIS). EIS is a measurement based on impedance, so EIS is inversely proportional to DPV whose measurement is based on the generated current. Impedance data are usually represented by a Nyquist plot with the dependence of real impedance (*Z*') on imaginary impedance (*Z*'') [54]. Characterization of SPCE-Au can be followed by EIS the changes in charge transfer resistance (*R*_ct_), resulting from the interaction of the analyte with the receptor [55]. *R*_ct_ can be evaluated from the semicircular portion of the Nyquist plot at higher frequencies. The increasing current due to electron transfer, the smaller the impedance and the smaller the *R*_ct_ diameter [56].

**Figure 5.**
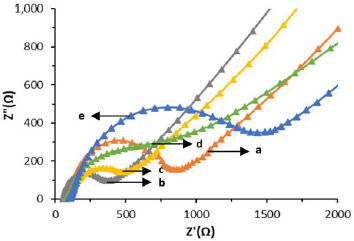
SPCE characterization results using electrochemical impedance spectroscopy: (a) SPCE Bare; (b) SPCE-Au; (c) SPCE-Au/Cysteamine; (d) SPCE-Au/Cysteamine/Glutaraldehyde; and (e) SPCE-Au/Cysteamine/Glutaraldehyde/Anti-ENaC with a redox system of 10 mM K_3_[Fe(CN)_6_] solution in 0.1 M KCl with a frequency of 0.1 Hz to 1000000 Hz at an anodic peak current potential of 0.01 V.

In the results of SPCE characterization with DPV it is known that there is an increase in current due to the process of electron transfer between the electrode and the analyte. Meanwhile, the results of the SPCE characterization using EIS (Figure 5) show that the larger *R*_ct_ diameter indicates a lower current resulting from electron transfer so that the resistance obtained is large. Table 2. shows a comparison of the measurement results of each stage of immunosensor modification using DPV and EIS.

### Morphological characterization of ENaC immunosensors with a scanning electron microscope

**Figure 6.**
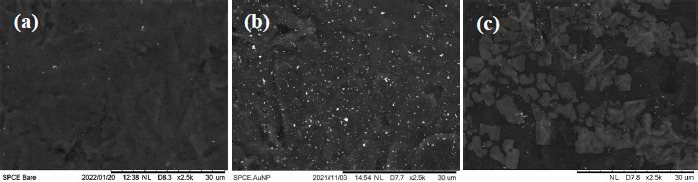
The modified SPCE characterization using SEM: **(a)** SPCE Bare, **(b)** SPCE-Au, and **(c)** SPCE-Au/Cysteamine/Glutaraldehyde/Anti-ENaC.

SPCE surfaces before and after being modified with gold nanoparticles were characterized using SEM. Figure 6 shows the SEM images of (a) SPCE Bare, (b) SPCE-Au, and (c) SPCE-Au/cysteamine/glutaraldehye/anti-ENaC.

The results of SPCE-Au characterization showed that after being modified with gold nanoparticles (SPCE-Au), the surface of the SPCE electrode became more closed due to the attached gold nanoparticles. This indicates that the AuNP has been evenly distributed on the entire surface of the electrode. Meanwhile, the SPCE-Au/cysteamine/glutaraldehyde/anti-ENaC characterization showed that the anti-ENaC immobilization had been successfully carried out, where antibody molecules were seen covering the electrode surface.

### Determination of optimum conditions with Box-Behnken experimental design

The optimum conditions of the immunosensor were determined by carrying out a Box-Behnken experimental design on the Minitab19 application with three factors optimized in the experiment, namely anti-ENaC concentration (X1), glutaraldehyde incubation time (X2) and anti-ENaC incubation time (X3). The effect of anti-ENaC concentration, glutaraldehyde incubation time, and anti-ENaC incubation time was tested using differential pulse voltammetry with a redox system of 10 mM K_3_[Fe(CN)_6_] solution in 0.1 M KCl at a potential range of -1.0 to 1 0.0 V at a scan rate of 0.008 V/s, *E*_step_ 0.004 V with an *E*_pulse_ of 0.025 V and a *t*_pulse_ of 0.05 s. Each factor is designed at three levels, namely the lowest level (-1), the middle level (0), and the highest level (+1), as shown in Table 3. The relationship between the response and the factors is determined through a sequence of experiments to obtain the optimum response results. Experiment with three factors and three different levels so as to give the number of trials 15 times. From the experimental current response obtained, the regression equation (2) is obtained as follows:


(4)

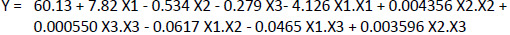



From equation (4), can be seen that a factor with a negative value reduces the response, while a factor with a positive value increases the response of the experimental stream. Based on the current response analysis, the ANOVA results are shown in Table 3 through the p-value, which explains the variability of the data. A *p*-value less than 0.05 indicates that a single variable exhibits a linear effect that is consistent with the linear model. The p-value is also used to determine the significance of each variable and the interaction effect shown by the combination of the two variables [44]. Based on the results of the ANOVA analysis shown in Table 3, it is known that all the optimized factors, namely the concentration of anti-ENaC, incubation time of glutaraldehyde, incubation time of anti-ENaC significantly affected the decrease in the current response, because it had a *p*-value <0.05. In table 3, can be seen that the p-value of each factor is X1 = 0.000, X2 = 0.001, and X3 = 0.0012.

**Figure 7.**
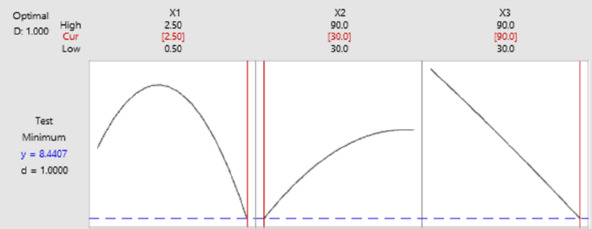
Response optimization of the optimal experimental conditional.

Response optimization needs to be done to identify setting variables and to optimize one or more responses. It is necessary to evaluate the number of responses to a variable [44]. Based on the results in Figure 7, the best optimization is close to the optimal state for the overall operating conditions of anti-ENaC concentration at 2.5 ng/mL, glutaraldehyde incubation time of 30 minutes, and anti-ENaC incubation time of 90 minutes.

### Determination of analytical parameters

After the optimum conditions of the parameters are known, a calibration curve is made and the detection and quantification limits of the developed ENaC immunosensor are calculated. The calibration curve for the ENaC immunosensor was made using various concentrations of the ENaC antigen tested on the immunosensor. The variation of the ENaC antigen concentration was made to 0.09375; 0.1875; 0.375; 0.75; 1; and 1.5 ng/mL.

**Figure 9.**
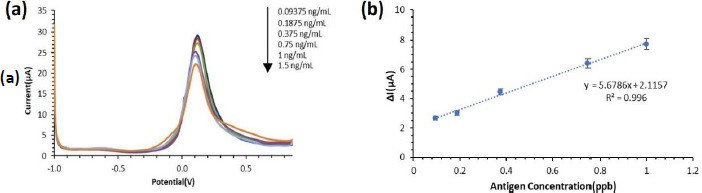
**(a)** The results of differential pulse voltammetry measurements for various concentrations of ENaC (0.09375; 0.1875; 0.375; 0.75; 1; 1.5) ng/mL with a redox system of 10 mM K_3_[Fe(CN)_6_] solution in KCl 0 .1 M over a potential range of -1.0 to +1.0 V, *E*_step_ 0.004 V, *E*_pulse_ 0.025 V, *t*_pulse_0.05 s, and scan rate 0.008 V/s. **(b)** ENaC immunosensor calibration curve with variations in concentration (0.09375; 0.1875; 0.375; 0.75; 1) ng/mL.

The response of the ENaC immunosensor current obtained was plotted against the response in the form of a reduction current difference measured three times for each antigen concentration. The ENaC immunosensor was characterized using differential pulse voltammetry (DPV) with a K_3_[Fe(CN)_6_] solution redox system at a potential range of -1.0 to +1.0 and a scan rate of 0.008 V/s. The voltammogram of the characterization results for each concentration of ENaC antigen is shown in Figure 9(a). Based on the results of the characterization, it can be seen that the higher the concentration of the ENaC antigen, the lower the current generated. The ENaC antigen is a large biomolecule that is not electroactive so when more ENaC antigen binds to the antibody on the electrode surface, the electron transfer process between the analyte and the electrode will be increasingly hindered.

Furthermore, the results of the characterization of the variation of the ENaC antigen concentration were plotted to create a calibration curve, as shown in Figure 9 (b), so that the linear regression equation was obtained, *y* = 5.6786*x* + 2.1157 with *R*^2^ = 0.996. The detection limit is 0.0372 ng/mL and the quantification limit is 0.124 ng/mL. In this study, the LoD obtained is lower than similar immunosensor studies as a method of detecting hypertension biomarkers, which can be seen in Table 4. This shows that the electrochemical immunosensor method using screen-printed carbon electrode modified with gold nanoparticles and a self-assembled monolayer can be used for the detection of ENaC protein as a biomarker of hypertension.

## Conclusions

Based on the results and discussion, it can be concluded that Anti-ENaC was successfully mobilized on the SPCE-Au surface. The optimum experimental conditions, obtained through the Box-Behnken experimental design, were anti-ENaC concentration of 2.5 g/mL, the incubation time of glutaraldehyde for 30 minutes, and the incubation time of anti-ENaC for 90 minutes. The performance of the immunosensor developed in this study resulted in a detection limit and a quantification limit of 0.0372 ng/mL and 0.124 ng/mL. The LOD obtained shows that this electrochemical immunosensor can differentiate ENaC protein levels in urine samples of non-hypertensive patients, without hypertension, or with a family history of hypertension, between 1.12 to 4.0 ng/mL or higher. This proves that the developed electrochemical immunosensor has the potential to be a point of care testing compared to the ELISA method in the detection of ENaC protein as a salt-sensitive biomarker of hypertension.

## Figures and Tables

**Figure 8.**
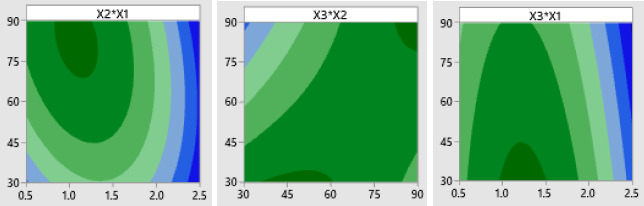
Contour plot of the experimental conditional.

**Table 1.** Optimization of the factors that affect the experimental conditions with the Box-Behnken design

Factor	Level		
	-1	0	+1
Anti-ENaC concentration, μg/mL	0,5	1,5	2,5
Glutaraldehyde incubation time, min	30	60	90
Anti-ENaC incubation time, min	30	60	90

**Table 2.** Comparison of the measurement results of the ENaC immunosensor using DPV and EIS.

SPCE Modification Stage	Current, μA	Resistance, Ω
SPCE Bare	31.355	0.767
SPCE-Au	77.298	0.290
SPCE-Au/Cysteamine	67.363	0.445
SPCE-Au/Cysteamine/Glutaraldehyde	47.375	1.027
SPCE-Au/Cysteamine/Glutaraldehyde/Anti-ENaC	31.866	1.150
SPCE Bare	31.355	0.767

**Table 3.** Analysis of variance.

Source	DF	Adj SS	Adj MS	*F*-value	*p*-value
Model	9	1291.72	143.524	64.99	0.000
Linear	3	1097.84	365.945	165.71	0.000
X1	1	975.76	975.759	441.84	0.000
X2	1	89.82	89.820	40.67	0.001
X3	1	32.26	32.257	14.61	0.012
Square	3	130.53	43.511	19.70	0.003
X1 X1	1	62.85	62.854	28.46	0.003
X2 X2	1	56.76	56.756	25.70	0.004
X3XX3	1	0.90	0.903	0.41	0.551
2-Way Interaction	3	63.35	21.117	9.56	0.016
X1 X2	1	13.69	13.686	6.20	0.055
X1 X3	1	7.77	7.770	3.52	0.120
X2 X3	1	41.89	41.893	18.97	0.007
Error	5	11.04	2.208		
Lack-of-Fit	3	9.63	3.211	4.56	0.185
Pure Error	2	1.41	0.704		
Total	14	1302.76			

**Table 4.** Electrochemical biosensors studies to detect epithelial sodium channel (ENaC).

Methods	Sensing method	Linear range, ng/mL	LOD, ng/mL	Reference
SPCE-Au/MPA/EDC-NHS	DPV and EIS	0,1 – 1,5	0,037	[14]
SPCE/Au/cysteamine/ bioconjugate	DPV and EIS	0,09375 – 1,0	0,084	[12]
SPCE/CeO2/streptavidin/aptamer	DPV and EIS	0,05 – 3,0	0,012	[53]
SPCE/RGO	DPV and EIS	0,09375 – 1,5	0,198	[13]
SPCE/CeO2/bioconjugate	DPV	0.047 – 3,0	0,110	[15]
SPCE-Au/cysteamine/glutaraldehyde	DPV and EIS	0,09375 – 1,0	0,0372	This work
